# Melorheostosis (Leri’s Disease): A Review

**DOI:** 10.7759/cureus.61950

**Published:** 2024-06-08

**Authors:** Nikita S Deshmukh

**Affiliations:** 1 Musculoskeletal Physiotherapy, Ravi Nair Physiotherapy College, Datta Meghe Institute of Higher Education & Research, Wardha, IND

**Keywords:** physical therapy, dripping candle wax, leri's disease, sclerosing bone disease, melorheostosis

## Abstract

Melorheostosis is a noncancerous bone disease characterized by abnormal bone and soft tissue growth. Despite being identified almost a century ago, there are still many unknown aspects surrounding this condition. It can often be an incidental discovery, with patients experiencing associated pain and deformities. Diagnosis typically relies on X-rays, although not all cases exhibit the classic candle wax appearance. A new imaging sign known as the “dumpling on a plate sign” has been proposed for flat bones for both MRI and CT scans. A biopsy may be necessary in cases of uncertainty, as there is not a definitive histological feature. It is not uncommon for melorheostosis to be linked with other conditions, and a collaborative approach involving a multidisciplinary team should be considered. This condition should be considered in the differential diagnosis of sclerotic bone conditions. Management is generally aimed at symptom relief, either through conservative measures or surgical intervention.

## Introduction and background

Melorheostosis, also known as Leri’s disease, is a rare bone condition with a wax-like appearance due to increased cortical bone formation. The name comes from the Greek words for limb and flow, describing the flowing hyperostosis in this condition. The prevalence of melorheostosis is approximately 0.9 cases per million people, with an estimated incidence of one in 1,000,000. This condition affects men and women equally and can occur in children and adults [1]. Melorheostosis typically manifests in the extremities. Interestingly, this condition is usually limited to one limb but rarely affects a single bone. It is not influenced by genetics or gender. While it can develop at any age, it commonly presents in teenagers and young adults. In childhood, the first signs may include contractures and deformities. Patients often experience mild to moderate pain. The overlying soft tissue may be underdeveloped, and the skin may resemble scleroderma [2]. The diagnosis of melorheostosis is typically made through a radiographic assessment, indicated by the “dripping candle wax” sign. Additional support can be obtained through normal serum calcium, phosphorus, and alkaline phosphatase levels, as well as anatomopathological tests that often reveal a combination of mature and immature bone in a dense structure with increased trabecular bone. Scintigraphy can also be utilized to indicate higher uptake. Treatment for melorheostosis is primarily symptomatic, involving the administration of analgesic or anti-inflammatory medications [3]. Localized pain can commonly occur as the primary symptom and, at times, the sole indicator observed. Concurrent skin issues, stiff joints, and variations in limb length can also be present. Typically, clinicians rely on clinical and imaging features to confirm the diagnosis. In adults, X-rays often reveal four distinct radiological patterns of melorheostosis: the recognizable “dripping wax” appearance, lesions resembling osteomas, myositis ossificans-like features, osteopathia striata-like characteristics, or a blend of these. While biopsies are usually unnecessary for diagnosis, they can be beneficial for cases with uncertain radiographic findings. Typical histological features of meloreostotic lesions include amplified cortical density, woven bone structures, increased osteoid buildup, and heightened vascularity. Melorheostosis frequently overlaps with conditions like osteopoikilosis and Buschke-Ollendorff syndrome [4].

## Review

Definition

Melorheostosis is an uncommon and advancing condition distinguished by the enlargement or thickening (hyperostosis) of the external layers of the bone (known as the cortical bone). This condition impacts the growth and development of both bone and soft tissues [5].

Location

Melorheostosis can manifest in various regions of the bone, with a higher predilection for affecting long bones, although it can potentially impact any part of the skeletal structure. Typically, it presents as localized and involves only one side. Involvement of soft tissues is exceedingly uncommon [1].

Pathogenesis and etiology

The cause of melorheostosis remains a mystery, with various theories proposed to explain the occurrence of this condition. Recent studies in molecular biology have identified mutations in the MAP2K1 gene, responsible for the dripping candle wax form, and mutations in SMAD3 for the endosteal form. It has been documented that melorheostosis occurring in isolation is related to random somatic mutations of the MAP2K1 gene, which encodes the MEK1 protein kinase involved in the RAS/MAPK signaling pathway [1]. The condition is attributed to mosaicism, resulting from postzygotic mutations leading to asymmetric skeletal structure involvement and accompanying soft tissue changes. Mutations in this pathway are often linked to the development of cancer. Furthermore, isolated MAP2K1 mutations can stimulate benign bone cell proliferation, causing melorheostosis. However, these mutations can disrupt the bone mineralization process, contributing to osteoid formation [3]. The MAP2K1 oncogene plays a crucial role in human bone formation and presents opportunities for future gene therapy in treating melorheostosis. The etiology of the disease remains unknown, with mosaicism proposed to explain its sporadic occurrence, segmental pattern, variable involvement extent, and equal gender distribution. Mutations in the LEMD3 gene have been identified in familial melorheostosis cases, indirectly linked to other dysplasias like osteopoikilosis [6]. Half of the affected patients may have MAP2K1 mutations, exhibiting classic radiological signs and higher osteoblast and osteoclast rates. Recent findings suggest somatic SMAD3 mutations can spur melorheostosis by enhancing the TGF-β/SMAD pathway. Various pathogenic factors, including developmental, ischemic, telangiectatic, hypervascularity, and infection, are associated with melorheostosis, often co-occurring with OPK or LEMD3 mutations [4,7]. The underlying pathophysiology of MAP2K1-positive melorheostosis might be explained by a gradual deterioration of bone microarchitecture, which subsequently triggers a periosteal reaction akin to osteomyelitis or trauma, ultimately leading to overall cortical outgrowth [8]. The TGF-β/SMAD pathway contributes to the pathogenesis of melorheostosis, playing a critical role in skeletal development and homeostasis. Aberrations in this pathway have been implicated in skeletal disorders like Marfan syndrome and Loeys-Dietz syndrome [5]. Based on past microscopic or clinical findings, Leri hypothesized that an infectious process might be involved. Other theories have suggested vasomotor neurosis leading to vessel obliteration due to local overactivity of the sympathetic nervous system and resulting ischemia, subperiosteal telangiectasia during limb development, endocrine disturbances, congenital injury, or embryonic defects. Inflammatory or degenerative changes in the vascular walls, resulting in oxidative stress and mesenchymal tissue metaplasia, were also proposed based on microscopic findings. One theory posits that melorheostosis is a congenital abnormality caused by a limb bud defect. This theory is supported by the distinct linear pattern of distribution along the long bone axes and anomalies in the surrounding tissues. The frequent sclerotomal distribution of lesions, corresponding with segmental sensory nerve supply, the high incidence of length discrepancies, and the coincidence with lesions in surrounding soft tissues further support the theory of a defect during embryonic development [9,10].

Associated conditions

The condition may present with various systemic manifestations. One patient displayed systemic high blood pressure, and subsequent examinations revealed a small kidney associated with melorheostosis. This condition may coexist with several other symptoms, like nephrotic syndrome, hyperpigmentation, linear scleroderma, thickening of subcutaneous tissue, capillary hemangioma, venous dilation, arteriovenous aneurysms, vascular nevus, fibroma, fibrolipoma, lipomatosis, and retroperitoneal fibrosis [11,12]. Soft tissue tumors, such as desmoid tumors and multicentric fibromatosis, are commonly found in the upper limbs. Benign bone tumors like intrathecal lipoma and fibro-lipomatous lesions primarily affect axial bones [13]. Additionally, cases of facial giant cell granuloma have been documented. It remains uncertain whether the presence of these malignant tumors is a result of transformation or a mere coincidence [14]. Archaeologists excavating ancient graves have observed this condition alongside diffuse idiopathic skeletal hyperostosis [15,16]. Sclerosing bone dysplasias are skeletal abnormalities that vary in severity and present a wide range of radiologic, clinical, and genetic features. Hereditary sclerosing bone dysplasias arise from disturbances in the pathways regulating osteoblasts or osteoclasts, leading to abnormal bone accumulation. Several genes have been identified that, when disrupted, cause specific types of hereditary sclerosing bone dysplasia, such as osteopetrosis, pyknodysostosis, osteopoikilosis, osteopathia striata, progressive diaphyseal dysplasia, hereditary multiple diaphyseal sclerosis, and hyperostosis corticalis generalisata. Many of these conditions exhibit similar pathological mechanisms involving endochondral or intramembranous ossification and share underlying genetic defects [17].

Clinical feature

The disease’s clinical manifestations can vary based on the affected site, the extent of bone involvement, and potential soft tissue involvement. Melorheostosis primarily affects young adults, typically in their second or third decade. While it can impact any bone, it commonly involves the lower limbs. The condition can present in various forms: monostotic (affecting a single bone), polyostotic (affecting multiple bones), or monomelic (affecting a single extremity) [18]. This diversity in symptom presentation poses a challenge for medical professionals approaching this condition. Melorheostosis may not show any symptoms and could be found incidentally during imaging for other reasons. Conversely, it can result in significant disability. Abnormal bone growth often affects soft tissues and reaches the joints, leading to a restricted range of motion (ROM) due to contractures and fibrosis. Soft tissue involvement can cause pain. Pain is usually localized to the affected joint, and the disease can sometimes mimic acute inflammatory arthritis, stiffness, and limited ROM in the affected joints, as well as complications like muscle weakness, contractures, and neuropathy in the surrounding tissues [5]. It is a benign dysplasia with a highly unusual and characteristic radiographic appearance [19]. The abnormal bone tissue growth in melorheostosis might compress or entrap nerves, causing neuropathic symptoms like tingling, numbness, and weakness in the affected areas. The severity of these symptoms varies based on nerve involvement and location [20]. Pain is a common symptom in melorheostosis patients, but this condition presents unique characteristics: it varies in intensity and location, can be dull or sharp, and is described as deep throbbing or burning [21]. The pain might be confined to or spread to nearby joints or soft tissues and may be sporadic or persistent, exacerbated by changes in climate or temperature [2]. Melorheostosis can also affect the skin, resulting in different dermatological signs, such as thickening of the skin over the affected bone with a rough or pebbly texture, referred to as dermal fibrosis [22].

Incidence

The rare condition affects fewer than one million people globally, with 40-50% of patients being diagnosed before the age of 20. While most literature indicates an even distribution between males and females, a recent study revealed a higher female prevalence of 4:1. Other case series have also shown a significant female predominance. Although any bone can be impacted, the lower limb is most affected. The disease primarily targets the diaphyseal and epiphyseal bones, sparing the axial bones and joints [22]. The condition can manifest as monostotic, polyostotic, monomelic, or hemimelic, with monomelic being the most frequent presentation. There have been reported instances of bilateral involvement of the upper limbs. Melorheostosis has no racial preferences, and while it progresses slowly in adults, it advances rapidly in children. When melorheostosis coexists with other sclerosing bone dysplasias, it is termed an overlap syndrome. The most commonly associated bone dysplasias include osteopoikilosis and osteopathic striata [15].

Investigations

The diagnosis of melorheostosis is typically established through radiological findings, including X-rays, CT, MRI, and bone scans. Each modality reveals specific features that have been well documented in various studies, providing a solid foundation for diagnosis. While a biopsy is performed in cases of suspicious or sinister lesions and often as part of surgical intervention, it is not mandatory for every case [23]. A bone scintigraphy scan of the entire body revealed consistent bone lesions exhibiting moderate radiotracer uptake, primarily late in the process. These lesions displayed a mix of sclerotic structures alongside lytic areas, thickened bone cortex, and signs of periosteal reaction [10]. Subsequently, an incisional biopsy was conducted using a specific method in the affected region, with histopathological analysis confirming a diagnosis of melorheostosis. Moreover, the findings were supplemented by immunohistochemical assessments following the conventional histopathological examination [7]. The typical radiographic appearance of melorheostosis features an irregular hyperostosis that affects the outer cortical bone. Often, this hyperostosis extends into the cancellous bone and can present as either a completely radiopaque or a mixed pattern. Commonly affected areas include the diaphysis of long bones, the pelvis, the ribs, and the bones of the hands and feet. Reports of craniofacial changes are less frequent. While the literature describes four distinct types of melorheostosis, the condition is more commonly classified as monostotic or polyostotic for practical purposes [24].

Surgical technique

Surgical operations encompass a variety of procedures, such as lengthening tendons, removing fibrous and bony tissue, releasing the fascia, adjusting joint capsules, performing bone cuts, relieving pressure on the spine and nerves, removing excessive bone growth, joint fusion, amputation, artificial joint placement, and procedures to elongate limbs. In children, corrective soft tissue release procedures may have a higher risk of failure, often requiring repetitive surgeries. Parents should be informed about the likelihood of procedures not being successful and, when possible, consider delaying surgery until the child reaches skeletal maturity. Core decompression has not proven beneficial, whereas distraction osteogenesis (callotasis) has shown favorable results in addressing differences in limb lengths. Incorrect placement of external fixation devices can lead to the need for multiple surgeries. Outside ankle deformities can be corrected using an external fixator. Surgeries are primarily conducted to alleviate symptoms [3,5].

Treatment

As noted previously, melorheostosis is a rare bone condition that stands as a primary source of pain and impairment, yet there are currently no medications available to modify the disease. Physical therapy (PT), consisting of regular exercise, presents numerous benefits compared to surgery and drug treatments, including easy implementation, minimal side effects, and relatively low expenses [25,26]. Hence, leading international organizations and experts universally recommend PT as a crucial treatment approach. Physical modalities are interventions that utilize physical stimuli such as electricity, heat, cold, or pressure to regulate pain signals. Depending on the specifics, like type, intensity, duration, and application site of the stimuli, physical modalities can impact the pain pathway at the peripheral, spinal, or supraspinal levels [11].

Pain Relief

Virtual reality (VR) therapy harnesses computer-generated environments to provide immersive experiences aimed at alleviating chronic pain. This type of therapy can potentially achieve pain relief through various mechanisms, such as cognitive distraction, visuotactile stimulation, and visuomotor stimulation. These combined effects might lead to neuroplastic changes by modifying sensory perception and influencing neural processing related to pain. Recent findings propose that VR therapy could potentially halt or reverse central sensitization, a pivotal mechanism in the shift from acute to persistent pain. On a molecular level, VR therapy seems to reduce the activation of NMDA receptors and the release of neuropeptides that contribute to spinal cord hyperexcitability, as indicated by animal studies. Furthermore, it appears to normalize increased levels of COX-2, TNF-α, and IL-1β linked to neuro-inflammation within pain pathways. At a cellular level, VR therapy lowers the firing of dorsal horn neurons and the development of wind-ups following repetitive stimuli. It also mitigates the long-term enhancement of synaptic communication between nociceptive afferents and spinal projection neurons. These findings suggest that VR therapy may impede the molecular and cellular processes that drive central sensitization [11].

Stiffness

In addressing symptoms characterized by stiffness and spasms, which can lead to limitations in ROM and motor function, a fundamental component of the PT regimen is the implementation of a self-stretching program. The incorporation of a stretching regimen holds promise for mitigating stiffness, thereby potentially enhancing ROM and motor function. An effective approach involves the application of targeted stretches to the affected area, with each stretch held for 30 seconds. This process is repeated three to four times during each session. The utilization of stretching techniques may yield positive outcomes by alleviating stiffness, consequently reducing pain, and facilitating smoother and swifter movement, thus fostering improvements in motor function [13]. It is emphasized that each stretch should be executed with deliberate slowness, ensuring a minimum hold period of 30 seconds. The recommendation for gentle and sustained stretching aligns with the findings advocated by Lorish et al., emphasizing the efficacy of prolonged stretching techniques in therapeutic interventions [27].

Contracture

While evidence supports interventions aimed at improving ROM, adhering to generally accepted principles is imperative to mitigate the impact or disability resulting from contractures. The following key concepts should guide the management of contractures:

Early prevention: Timely diagnosis and the initiation of physical medicine approaches, such as passive ROM exercises and splinting, are crucial to prevent contractures before their onset or during the early stages when they are mild [28].

Inevitability of contractures: In certain conditions, the development of contractures is unavoidable due to underlying factors.

Response to intervention: Contractures that have progressed to an advanced stage may become fixed and exhibit limited responsiveness to conservative measures like stretching or splinting, potentially necessitating surgical intervention.

Preservation of independent movement: A primary objective in managing contractures is to minimize their detrimental effects on autonomous movement.

Role of static positioning: Prolonged static positioning contributes significantly to forming contractures, underscoring the importance of regular repositioning and dynamic movement.

Impact of mild contractures: While severe contractures can significantly impair function, mild contractures may not exert a substantial negative impact on overall functionality.

Adhering to these principles can facilitate a comprehensive approach to managing contractures, thereby promoting optimal outcomes in terms of mobility and function [29].

Rehabilitation Management

To prevent or delay the onset of contractures in individuals at risk of musculoskeletal deformity, it is essential to adhere to a regimen comprising four primary PT modalities. These modalities encompass the following:

Regular periods of daily activity: Consistent engagement in prescribed periods of daily activity is fundamental for maintaining mobility and preventing immobility-related complications.

Passive stretching: Regular passive stretching of muscles and joints is crucial for preserving or enhancing the ROM and averting the development of contractures.

Limb positioning: Strategic positioning of the limbs is vital to facilitate movement and counteract restrictions that may lead to contractures.

Splinting: The utilization of splints serves as a valuable measure in preventing or delaying the onset of contractures by providing support and maintaining optimal joint alignment.

Passive stretching, in particular, plays a significant role in preventing contractures. Research has demonstrated its efficacy in impeding the progression of contractures. Initiation of a passive stretching program early in the course of treatment is paramount, incorporating regular sessions into both morning and evening routines. Employing proper technique is essential for maximizing the effectiveness of passive stretching exercises. Each stretch should be held for 15 seconds, with 10 to 15 repetitions performed per session, executed slowly and gently to avoid discomfort and ensure patient cooperation. To supplement verbal instructions and demonstrations provided by the physical therapist, written instructional materials should be furnished to patients and their families. The selection of stretching exercises tailored to specific anatomical regions will vary based on the underlying neuromuscular disease, necessitating individualized treatment approaches.

Muscle Weakness

In addressing muscle weakness, a regimen comprising strengthening exercises initially without weights, progressing to include weights, holds promise for mitigating this condition. Additionally, electrical muscle stimulation has demonstrated efficacy in improving muscle weakness. Our study encompasses investigations examining neuromuscular electrical stimulation (NMES) programs consisting of multiple sessions, administered either independently or as an adjunct to other forms of exercise. The NMES application targeted the affected muscle(s) either exclusively or in conjunction with additional muscle groups, such as the hamstrings, gastrocnemius, and glutei. Variability across programs was anticipated regarding stimulation parameters, including frequency (Hz), pulse type and width (µs), duty cycle (expressed as a percentage of active intervention time), session duration (minutes), frequency (sessions per week), and overall program duration (weeks). Studies focusing solely on the acute effects of NMES following a single session were excluded. No restrictions were imposed regarding the site of stimulation or the specific parameters utilized. Interventions were compared against either inactive controls (e.g., no treatment, placebo, or sham NMES) or active controls, such as alternative forms of exercise [30].

## Conclusions

Despite significant advancements in medicine, considerable challenges persist in addressing melorheostosis, a condition that has remained largely unchanged over the past century. Due to its rarity, the available literature primarily consists of case reports or case series, reflecting a limited understanding of the condition. The rarity and lack of awareness surrounding the condition contribute to its low prevalence, with only a few hundred cases reported in the medical literature. This rarity means that many healthcare professionals might never encounter a case in their careers. Due to its rarity, there is limited exposure to the condition during medical training, resulting in limited knowledge. This lack of familiarity can lead to misdiagnosis or delayed diagnosis. The non-specific symptoms and varied presentation of melorheostosis can include pain, stiffness, deformity, and restricted movement. These symptoms overlap with many other, more common musculoskeletal conditions. The progressive nature of this condition means that symptoms develop slowly, making it harder to recognize early and distinctly. Diagnostic challenges arise due to radiological similarities between melorheostosis and other conditions. Despite melorheostosis having distinctive radiological features, such as the “dripping candle wax” appearance, confusion with conditions like osteoma, osteopathia striata, or osteosarcoma may occur, particularly if the radiologist is not specifically looking for melorheostosis. The lack of specific blood tests or biomarkers for melorheostosis limits the ability to use simple diagnostic tests to confirm the condition. The diagnosis is primarily radiological and clinical, relying heavily on imaging. The etiology of melorheostosis remains idiopathic, and while radiological imaging aids in diagnosis, there is a notable absence of a formal classification system for the condition. Instead, only radiological patterns have been identified. Melorheostosis should be considered in cases involving bone sclerosis, highlighting the importance of diagnostic vigilance. Management strategies primarily focus on providing symptomatic relief, as no pharmacological interventions capable of curing the disease have been identified to date. Thus, despite medical advancements, melorheostosis continues to pose significant challenges in both diagnosis and treatment.
